# Mechanical Properties and Atomic Explanation of Plastic Deformation for Diamond-Like BC_2_

**DOI:** 10.3390/ma9070514

**Published:** 2016-06-24

**Authors:** Baobing Zheng, Meiguang Zhang, Shaomei Chang

**Affiliations:** College of Physics and Optoelectronics Technology, Nonlinear Research Institute, Baoji University of Arts and Sciences, Baoji 721016, China; zhmgbj@126.com (M.Z.); csm7027@163.com (S.C.)

**Keywords:** ideal strengths, ab initio calculations, anisotropic properties, boron-carbon compound

## Abstract

Motivated by a recently predicted structure of diamond-like BC_2_ with a high claimed hardness of 56 GPa (*J. Phys. Chem. C* **2010**, *114*, 22688–22690), we focus on whether this tetragonal BC_2_ (*t*-BC_2_) is superhard or not in spite of such an ultrahigh theoretical hardness. The mechanical properties of *t-*BC_2_ were thus further extended by using the first principles in the framework of density functional theory. Our results suggest that the Young’s and shear moduli of *t-*BC_2_ exhibit a high degree of anisotropy. For the weakest shear direction, *t-*BC_2_ undergoes an electronic instability and structural collapse upon a shear strain of about 0.11, with its theoretically ideal strength of only 36.2 GPa. Specifically, the plastic deformation under shear strain along the (110)[001] direction can be attributed to the breaking of *d*1 B–C bonds.

## 1. Introduction

Owing to the great demand from mechanical machining and semiconductor industries, the experimental and theoretical attempts to synthesizing and designing superhard materials have been performed intensively in recent decades. Although diamond is the hardest material with a Vickers hardness of 115 GPa along the (111) plane, the shortcoming of its poor resistance to oxidation as well as the reaction with ferrous metals restrict the applications of diamond. Those limitations have stimulated the continuous quest for novel superhard compounds with better thermal and chemical stabilities than pure diamond. In addition to possessing advanced electrical and optical properties as well as high hardness, the boron–carbon compounds have proven to be more highly resistant to oxygen and ferrous metals than similar carbon materials, which thus are unexceptionable substitutions for pure diamond. The typical B–C compound is boron-rich boron carbide (B_4_C), which is characterized by a unique combination of properties, prompting it as a choice of engineering material [1]. On the other hand, the boron-doped diamond lattice, which forms carbon-rich B–C material, changes it from an insulator into a *p*-type semiconductor with boron acting as a charge acceptor. Such fascinating electrical and mechanical properties has spurred researchers on to devote much effort to those carbon-rich B–C systems (BC_2_, BC_3_, BC_5_, and BC_7_) [2,3,4,5,6,7,8,9].

Recently, Zinin et al. synthesized a cubic BC_3_ phase with an *sp*^3^ bonding network at a pressure of 39 GPa and a temperature of 2200 K [4]. Solozhenko et al. had reported the synthesis of diamond-like BC_5_ with the highest boron content ever achieved under high pressure and high temperature condition [5]. The synthesized phase exhibits extreme Vickers hardness (71 GPa) and high thermal stability (up to 1900 K), which makes cubic BC_5_ an exceptional superabrasive overcoming diamond. However, the determinations of the synthesized B–C crystal structures are still open questions due to the similarity in both electronic and nuclear scattering cross sections for boron and carbon [1,10,11]. Theoretically, first-principle calculations combined with a state-of-the-art structure prediction have emerged as a powerful approach that complements experiments and achieved great success in the crystal structure prediction, especially in extreme physical conditions. Based on hypothetical configurations method and an ab initio evolutionary algorithm, Xu et al. [6] predicted a tetragonal lattice structure (space group *I*4_1_/*amd*, No. 141) for BC_2_ (*t*-BC_2_) whose mechanical and dynamical stability have been confirmed by the criterions of elastic constants and phonon frequencies. Despite possessing the high theoretical Vickers hardness (56 GPa), we should carefully debate whether *t-*BC_2_ is superhard. The main reasons can be summarized as follows: (1) the absence of uniform and accurate hardness formula usually leads to the contradictory results for the same crystal for different models of hardness; and (2) the hardness is generally measured at finite strains where bonding characteristics of materials may change significantly [12], whereas the theoretical hardness estimated by the parameters at the equilibrium structure is thus not suitable to account for the mechanical strength of materials. Indeed, the ideal tensile and shear strengths of material evaluated when the lattice becomes unstable, which is the upper bound on the mechanical strength, are therefore more appropriate and stringent for the reflection of hardness than elastic parameters. In particular, the ideal shear strength describes the shear resistance of the system at the atomic level where plastic deformation occurs [13], so the ultimate hardness of a material may be assessed from its ideal shear strength and bonding nature, and this fact has been verified by a number of previous studies [14,15,16,17,18].

In the present paper, we perform the first-principles calculations to further investigate the structural and mechanical properties of the recently predicted *t-*BC_2_. The obtained orientation dependences of the Young’s and shear moduli are illustrated to show the mechanical anisotropy for *t-*BC_2_. The ideal tensile and shear strengths of *t-*BC_2_ are also estimated to provide a deeper insight into mechanical behavior and hardness.

## 2. Computational Methods

The total energy calculations were performed using density functional theory with the Perdew–Burke–Ernzerhof (PBE) exchange correlation in the framework of the generalized gradient approximation (GGA) as implemented in the Vienna ab initio simulation package (VASP) [19,20]. The electron and core interactions were described by the projector augmented-wave (PAW) method combined with the frozen core approximation [21], and the 2*s*^2^2*p*^1^ and 2*s*^2^2*p*^2^ were considered as valence electrons for B and C, respectively. The plane-wave basis set was truncated with an energy cutoff of 800 eV, and the Brillouin zone integration was generated using Monkhorst-Pack *k* point meshes [22] with a grid of 0.03 Å^−1^ and 0.025 Å^−1^ for total-energy and elastic constants calculations, respectively, which ensure that the enthalpy results were well converged to below 1 meV/f. u. The elastic constants were calculated by the strain–stress method, which has been successfully utilized previously [23,24]. The bulk modulus, shear modulus, Young’s modulus, and Poisson’s ratio were estimated via Voigt–Reuss–Hill approximation [25]. To obtain the strain–stress relationships and ideal strengths, *t-*BC_2_ cell was deformed continuously by increasing the displacement in the direction of the applied strain [15,26]. In addition, the 3D electron localization function distributions are illustrated by VESTA [27].

## 3. Results and Discussion

**Figure 1:**
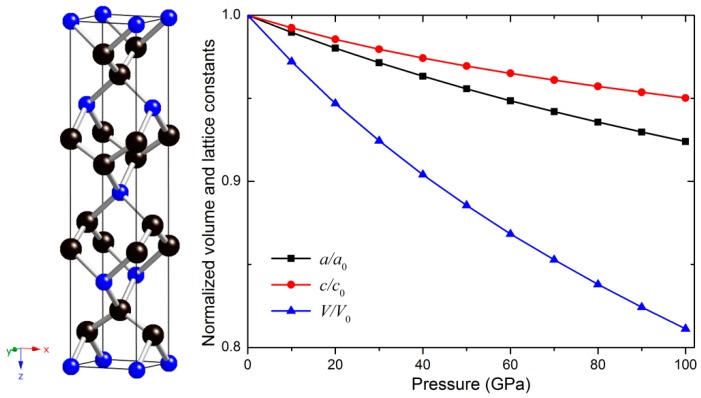
The crystal structure and the dependence of normalized lattice constants and cell volume on pressure for *t-*BC_2_. The blue and black spheres denote B and C atoms, respectively.

The crystal structure, as well as the dependences of the normalized lattice parameters and volume on pressure up to 100 GPa for *t-*BC_2_, is shown in Figure 1. The equilibrium lattice parameters for *t-*BC_2_ are *a* = 2.5227 Å and *c* = 11.9373 Å, which are in excellent agreement with the evaluated results reported in [6]. The optimized *t-*BC_2_ structure holds a tetragonal lattice with 12 atoms, and the two nonequivalent B and C atoms occupy the Wyckoff 4*a* (0.0, 0.0, 0.0) and 8*e* (0.0, 0.0, 0.34085) positions, respectively. The calculated bond lengths of C–C and B–C bonds are 1.502 Å and 1.663 Å, which are slightly different from the values (1.500 Å and 1.662 Å) proposed in [6], respectively. The estimated density is 3.06 g/cm^3^, which is lower than that of diamond (3.52 g/cm^3^). The remarkable consistency between the calculated results and [6] completely confirms the accuracy and reliability of the present calculations. The positive formation energy of *t-*BC_2_ (0.491 eV/atom), defined as $E_{f}=E_{\text{tot}}\left({\text{BC}}_{2}\right)-E\left(\text{solid B}/\text{atom}\right)-2E\left(\text{diamond}/\text{atom}\right)$, suggests that *t-*BC_2_ is meta-stable at ambient conditions. Compared with the BC_3_ structures synthesized experimentally, *t-*BC_2_ is more stable due to the greater formation energy of the BC_3_ (0.580 eV/atom). The calculated total energy of *t-*BC_2_ is −8.133 eV/atom, higher than that of the diamond-like BC_3_ (−8.351 eV/atom) and graphitic BC_3_ (−8.402 eV/atom) phases [28].

The estimated elastic constants, bulk modulus *B*, shear modulus *G*, and Young’s modulus *E* for *t-*BC_2_ are listed in Table 1 with the theoretical and experimental results of some previous B–C–N compounds for comparisons. For a stable tetragonal structure, the six independent elastic constants *C*_11_, *C*_12_, *C*_13_, *C*_33_, *C*_44_, and *C*_66_ should satisfy the necessary and sufficient Born–Huang elastic stability criteria as follows [37]:

(1) $$\begin{array}{l} C_{11}>\left|C_{12}\right|,\;2C_{13}^{2}<C_{33}(C_{11}+C_{12}),\\ C_{44}>0,\;C_{66}>0. \end{array}$$

Obviously, the calculated elastic constants of *t-*BC_2_ meet all the stability criteria, demonstrating the mechanical stability of *t-*BC_2_ at ambient pressure. Furthermore, the dependences of normalized lattice parameters on pressures up to 100 GPa for *t-*BC_2_, shown in Figure 1, indicate that *t-*BC_2_ structure along the *c*-axis is more incompressible than along the *a*-axis, which coincides with the result of elastic constants (*C*_33_ > *C*_11_). The compressibility of *t-*BC_2_ along the *x*- and *z*-directions as a function of pressure can be fitted by the least square method according to the lattice parameters and pressures, and the corresponding formulas are listed as follows:

(2) $$\frac{a}{a_{0}}=0.99958-1.00\times 10^{-3}P+2.51\times 10^{-6}P^{2};$$

(3) $$\frac{c}{c_{0}}=0.99941-7.14\times 10^{-4}P+2.27\times 10^{-6}P^{2}.$$

Typically, the hard material should possess a high bulk modulus to resist the volume deformation and a high shear modulus to support the shear deformation and thus enhance the resistance ability of material upon compression load. The calculated bulk modulus of *t-*BC_2_ is 333 GPa, much lower than that of diamond (432 GPa) but comparable to those of *c*-BN (376 GPa), *d*-BC_3_ (349 GPa), *dl*-BC_3_ (391 GPa), and *dl*-BC_5_ (376 GPa), suggesting that the *t-*BC_2_ phase is a greatly incompressible material. Physically, the shear modulus is more appropriate to evaluate the hardness of a material than the bulk modulus because the hardness tests measure plastic deformation of the material that appears to be closely linked to the deformation of a shear character [38]. Clearly, the shear modulus *t-*BC_2_ is 285 GPa, which is significantly smaller than that of superhard B–C–N compounds, such as diamond (517 GPa), *c*-BN (390 GPa), *d*-BC_3_ (318 GPa), *dl*-BC_3_ (344 GPa), and *dl*-BC_5_ (394 GPa). Compared with the diamond-like BC_x_ phases of low pressure synthesis (i.e., *dllp*-B_2_C_3_ and *dllp*-BC_4_), the shear modulus of *t-*BC_2_ is much larger than those of the two phases (170 GPa and 19.3 GPa, respectively). Note that the bulk and shear moduli of B–C systems gradually decrease with the increasing concentrations of boron, which is consistent with the results of [30]. Therefore, we should carefully reappraise whether *t-*BC_2_ with large boron concentrations is superhard. According to Pugh’s criterion, the calculated result of *G*/*B* for *t-*BC_2_ is 0.847, larger than 0.571, indicating its brittle mechanical properties.

**Figure 2:**
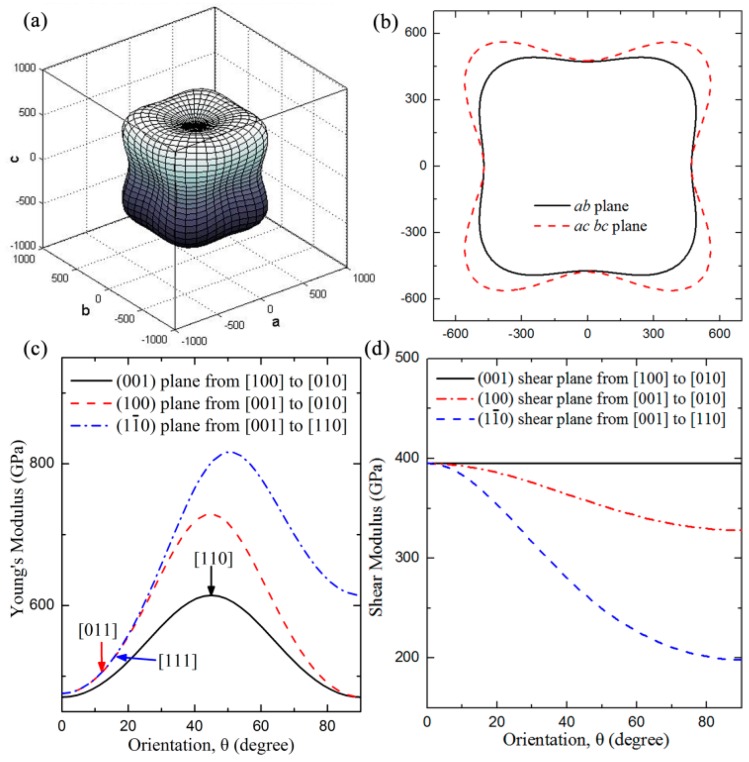
(**a**,**c**) Orientation dependence of Young’s modulus *E* and (**b**) the corresponding projection in *ab*, *ac*, and *bc* planes; (**d**) for *t-*BC_2_,orientation dependence of the shear modulus of *t-*BC_2_.

The mechanical anisotropy is the mechanical property of being directionally dependent, which can exert great influence on the properties of a physical mechanism. For a tetragonal structure, Young’s modulus for a tensile stress along an arbitrary [*hkl*] direction can be expressed as the following equation [39]:

(4) $$E^{-1}=s_{11}(\alpha^{4}+\beta^{4})+s_{33}\gamma^{4}+2s_{12}\alpha^{2}\beta^{2}+2s_{13}(\beta^{2}\gamma^{2}+\alpha^{2}\gamma^{2})+s_{44}(\beta^{2}\gamma^{2}+\alpha^{2}\gamma^{2})+s_{66}\alpha^{2}\beta^{2},$$

where *α*, *β*, and *γ* are the direction cosines of the tensile stress direction deduced from the transformed coordinate system with respect to the original coordinate system, and *s*_11_, *s*_12_, *s*_13_, *s*_33_, *s*_44_, and *s*_66_ are the independent elastic compliance constants given by Kelly et al. [39], which can be determined from the calculated elastic constants *C_ij_*. The shear modulus *G* on the (*hkl*) shear plane with shear stress applied along the [*uvw*] direction is given by

(5) $$\begin{array}{l} G^{-1}=4s_{11}\left(\alpha_{1}^{2}\alpha_{2}^{2}+\beta_{1}^{2}\beta_{2}^{2}\right)+4s_{33}\gamma_{1}^{2}\gamma_{2}^{2}+8s_{12}\alpha_{1}\alpha_{2}\beta_{1}\beta_{2}+s_{66}{\left(\alpha_{1}\beta_{2}+\alpha_{2}\beta_{1}\right)}^{2}\\ +\;8s_{13}\left(\beta_{1}\beta_{2}\gamma_{1}\gamma_{2}+\alpha_{1}\alpha_{2}\gamma_{1}\gamma_{2}\right)+s_{44}\left[{\left(\beta_{1}\gamma_{2}+\beta_{2}\gamma_{1}\right)}^{2}+{\left(\alpha_{1}\gamma_{2}+\alpha_{2}\gamma_{1}\right)}^{2}\right] \end{array},$$

where (*α*_1_, *β*_1_, *γ*_1_) and (*α*_2_, *β*_2_, *γ*_2_) are the direction cosines of the [*uvw*] and [*HKL*] directions in the primitive coordinate system, respectively, and [*HKL*] directions denote the vector normal to the (*hkl*) shear plane. To gain deeper insight into the mechanical anisotropy of *t-*BC_2_, the three-dimension plots of the Young’s modulus as a function of the crystal orientation and its projections onto the (*ab*), (*bc*), and (*ac*) crystal planes are shown in Figure 2a,b, respectively. The distance between the origin of the coordinate and the surface or profile of the graph denotes the value of Young’s modulus along a certain direction. The large difference between the obtained shape and the sphere of a perfect isotropic crystal for the three-dimensional plots of Young’s modulus reveals that *t-*BC_2_ presents a high degree of anisotropy and hence is easily deformed along a certain direction under strain. Figure 2c,d illustrate the orientation dependence of Young’s modulus and shear modulus, respectively. From Figure 2c, we can clearly see that the maximum Young’s modulus (816 GPa) is 73.2% larger than the minimum Young’s modulus (471 GPa), further suggesting the high degree of anisotropy for *t-*BC_2_. The sequence of Young’s modulus along principle crystal orientations is summarized as follows: *E*_[010]_ < *E*_[001]_ < *E*_[011]_ < *E*_[111]_ < *E*_[110]_. As plotted in Figure 2d, the shear modulus along the (001) plane is independent of shear stress directions since the analytical result of shear modulus along this plane is described as *G* = 1/*s*_44_ = *C*_44_ = 395 GPa. The shear moduli along the (100) and ($1\bar{1}0$) planes decrease gradually with the increase in the angle of orientation, and the minimum shear modulus (199 GPa) distributes along the [110] direction within the ($1\bar{1}0$) basal plane.

**Figure 3:**
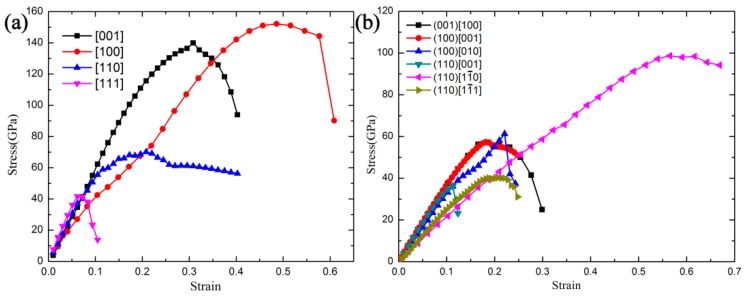
Calculated strain–stress relations for *t-*BC_2_ in various tensile (**a**) and shear (**b**) directions.

Despite possessing ultrahigh elastic modulus, the feature of superhard for *t-*BC_2_ needs further confirmation, because the high values of elastic moduli can only represent high elastic stiffness—not a high degree of plastic harness [13]. Physically, the ideal strength, which is the ability to withstand an applied load up to yield, is more suitable to measure the hardness than the elastic modulus. The main reason is that the elastic modulus is a measure of elastic response that is non-permanent, and the material will return to its original shape when the applied load is removed, but the measurement of hardness is accompanied with plastic deformation involving the breaking of atomic bonds. To evaluate the strength determined by bond strength and the breaking nature under strain and essentially explore the atomistic origin of the structural deformation mechanisms, we calculated the ideal tensile and shear strengths of *t-*BC_2_ by means of applying a series of continuous strains along a specified direction, the calculated results are illustrated in Figure 3a,b. The ideal tensile strengths along the [001], [100], [110], and [111] directions are 139.9, 152.1, 70.1, and 41.6 GPa, respectively. Note that all the tensile strengths are larger than 40 GPa, and the minimum tensile strength occur in the body-diagonal [111] direction, which can be attributed to the absence of B–C and C–C bonds along the body-diagonal direction. Compared with the typical cubic BC_3_ (*d*-BC_3_) [31], the ideal tensile strength of *t-*BC_2_ along the [110] and [111] directions are slightly lower than those of the *d*-BC_3_ (σ_[110]_ = 77.6 GPa, σ_[111]_ = 52.5 GPa). Strikingly, the ideal tensile strength of *t*-BC_2_ along the [001] directions is even larger than that of the superhard *d*-BC_3_ (σ_[001]_ = 107.6 GPa).

**Figure 4:**
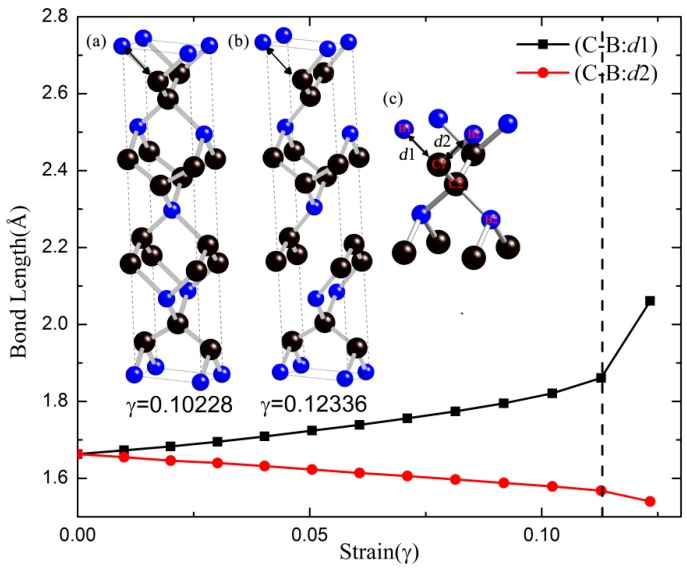
Calculated bond lengths as a function of strain for *t-*BC_2_ under shear deformation along the (110)[001] directions. Insets: crystal structures before (**a**) and after (**b**) shear instability, and basic building blocks in *t-*BC_2_ (**c**). The dashed line represents the shear-induced structural deformation’s first occurrence.

The shear stress responses in the (001), (100), and (110) planes were evaluated to search the easy cleavage plane of *t-*BC_2_. From Figure 3b, we can conclude that the ideal shear strengths show a high degree of anisotropy along the different shear directions, and the largest ideal shear strength in the $\left(110)[1\bar{1}0\right]$ shear direction (98.6 GPa) is 2.7 times larger than the lowest ideal shear strength in the (110)[001] shear direction (36.2 GPa). The minimum of the ideal shear strength is 13.0% lower than the tensile strength along the [111] direction, suggesting that the failure mode in *t-*BC_2_ is dominated by the shear type. To obtain the fundamental mechanism of plastic deformation along the weakest shear direction, the bond lengths of *d*1 and *d*2 B–C bonds as a function of the shear strains in the (110)[001] direction are plotted in Figure 4. The development of the structure for *t-*BC_2_ under shear deformation along this direction and the local bonding structure are also sketched by the inset a, b, and c in Figure 4. Clearly, the *d*1 and *d*2 B–C bonds are identical at equilibrium (shear strain *γ* = 0). Then, with the increase of shear strain, the *d*1 B–C bond declines slightly but the *d*2 B–C bond rises rapidly. Once the shear strain goes beyond the critical strain (*γ* = 0.11279), the *d*2 bond increases to 2.061 Å abruptly, indicating the breaking of the *d*2 bond and the instability of the *t-*BC_2_ structure. In addition, the behavior of the covalent *d*2 bond breaking can be observed from the selected crystal structures before (inset a) and after (inset b) shear instability in Figure 4.

**Figure 5:**
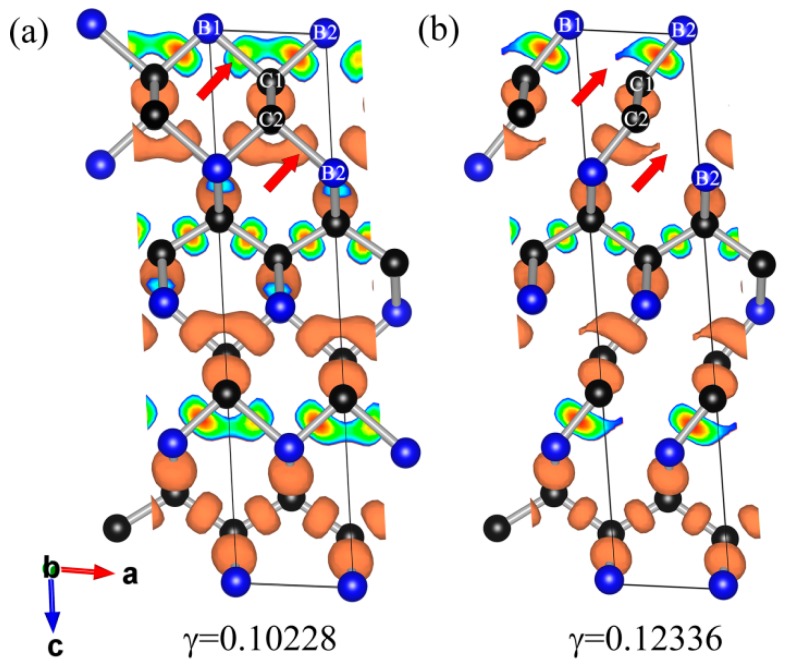
Developments of ELF for *t-*BC_2_ during shear in the (110)[001] slip before (**a**) and after (**b**) shear instability.

Note that the lowest ideal strength (36.2 GPa) is much lower than the calculated hardness (56.0 GPa [6]) estimated by Guo’s hardness formula [40]. Compared with typical boron oxide, the experimental Vickers’ hardness of superhard boron suboxide B_6_O (i.e., 38 GPa), harder than other boron oxides [41], is in excellent agreement with ab initio density functional theory of strain–stress curves for B_6_O (the lowest ideal shear strength of 38 GPa) [13,41,42], suggesting that the ideal shear strength is the most appropriate mechanical property for reflecting the hardness of materials. However, the calculated lowest ideal strength of *t-*BC_2_ shows a large discrepancy with the estimated hardness according to Guo’s hardness formula. The main reason for this discrepancy is that this hardness formula is correlated with bulk *B* and shear *G* moduli, which are estimated by the equilibrium structure, but the ideal shear strength focuses on the maximum shear stress at the atomic level where the crystal is far from equilibrium structure. To further clarify this disparity, the three-dimensional isosurfaces of the electron localization function (ELF) before and after the shear instability with an isovalue of 0.75 (a typical good number for characterization of covalent bondings) are plotted in Figure 5a,b, respectively. Apparently, the high electron localization appears between all adjacent B and C atoms when the shear strain is lower than the critical value of 0.11279. However, once the shear strain exceeds the critical value, the electron localization of B1–C1 and B2–C2 bonds vanish (denoted by red arrows), as illustrated in Figure 5b. The chemical bond defined by Bader is characterized by the bond critical point (BCP) whose charge density and Laplacian value reflect the type of chemical bond and the bond strength. Thus, we performed the Bader charge analysis, and the corresponding results are summarized in Table 2. Due to the ultrahigh bond strength, the C–C bond of *t-*BC_2_ exhibit large values of the electron density ρ and a relatively negative Laplacian value, $\nabla^{2}\rho$, at the bond critical point, characteristic of primarily covalent bonding, which are comparable with those of diamond (ρ=1.60 eÅ^−3^ and $\nabla^{2}\rho =-15.24$ eÅ^−5^ [43]), suggesting the high bond strength of the C–C bond in *t-*BC_2_ and hence hardly producing shear failure. However, the charge density at the *d*1 B–C bond critical point decrease significantly as the increase of shear strain, the Laplacian value of the *d*1 B–C bond critical point changes from −4.717 to −0.606, indicating the weakening covalent *d*1 bond under the shear strain and thus breaking beyond the critical shear strain. It can be concluded that the structural failure under shear deformation along the (110)[001] shear direction is rationalized by the breaking of the *d*1 bond. Furthermore, the minimum ideal strength of 36.2 GPa, lower than 40 GPa (the criterion of superhard material), indicates that the plastic deformation would take place along the crystal plane (110), leading to electronic instability and the structural collapse after a shear stress of 36.2 GPa.

## 4. Conclusions

In summary, we have investigated the mechanical anisotropy and strengths of *t-*BC_2_ by using first-principles calculations. The calculated Young’s and shear moduli of *t-*BC_2_ present a high degree of anisotropy and hence easily lead to structural failure. The shear strength for the slip system (110)[001] is the lowest among all the six-slip system, and this slip system leads to the breaking of the *d*1 B–C bonds in *t-*BC_2_, supported by the results of ELF and BCP calculations. These results demonstrate that *t-*BC_2_ tends to undergo an electronic instability with a shear stress lower than 40 GPa for the (110)[001] slip system. It should be stressed that the simplified models of hardness by either semi-empirical or ab initio methods are not appropriate for measuring the hardness of materials, because these theories usually introduce some parameters (such as bond energetics and electron density) that are based on the equilibrium structure, whereas plastic deformation in crystals occurs far from equilibrium upon bond breaking in the practical measurement of hardness. Thus, the ideal shear strength derived from the strain–stress curve, which is relevant for plastic deformation, is most appropriate for measuring the hardness of materials. The present work provides fundamental information for a better understanding of the structural stability and mechanical performance of this interesting material.

## Figures and Tables

**Table 1:** Calculated elastic constants *C_ij_*, bulk modulus *B*, shear modulus *G*, and Young’s modulus *E* (in units of GPa). Also shown is *G*/*B* ratio.

Compounds	Source	*C*_11_	*C*_12_	*C*_13_	*C*_33_	*C*_44_	*C*_66_	*B*	*G*	*E*	*G*/*B*
*t*-BC_2_	Present	571	173	226	612	395	324	333	282	659	0.847
B_4_C_4_	Theory ^1^	656	191	167	562	311	382	324	285	660	0.879
*dl-*BC*_3_*	Theory ^2^	720	206	220	788	464	268	391	344	798	
*d*-BC_3_	Theory ^3^	658	195			393		349	318	731	
*dl*-BC_5_	Theory ^4^	818	156			442		376	394	876	
B_4_C	Experiment ^5^							240	193	456	
	Theory ^6^	562	124	70	518			234			
*c*-BN	Theory ^7^	786	172			445		376	390		
Diamond	Theory ^8^	1052	122			555		432	517		

^1^ [17]; ^2^ [29,30]; ^3^ [31]; ^4^ [32]; ^5^ [33]; ^6^ [34]; ^7^ [35]; ^8^ [32,36].

**Table 2:** Bond critical point data of *t-*BC_2_ structure before and after shear instability. *L* is the bond length. $\rho \left(r_{CP}\right)$ and $\nabla^{2}\rho \left(r_{\text{CP}}\right)$ are the charge density and its Laplacian at the corresponding critical points.

Bond	*γ* = 0.10288			*γ* = 0.12336		
	L(Å)	$\rho \left(r_{CP}\right)$	$\nabla^{2}\rho \left(r_{CP}\right)$	L(Å)	$\rho \left(r_{CP}\right)$	$\nabla^{2}\rho \left(r_{CP}\right)$
B1–C1(*d*1)	1.821	0.733	−4.717	2.061	0.461	−0.606
B2–C1(*d*2)	1.579	1.128	−1.800	1.540	1.254	−1.906
C1–C2(*d*3)	1.500	1.731	−16.179	1.485	1.791	−17.796
B2–C2(*d*4)	1.821	0.733	−4.746	2.061	0.461	−0.606
